# Flavonoids as Potential anti-MRSA Agents through Modulation of PBP2a: A Computational and Experimental Study

**DOI:** 10.3390/antibiotics9090562

**Published:** 2020-08-31

**Authors:** Hani A. Alhadrami, Ahmed A. Hamed, Hossam M. Hassan, Lassaad Belbahri, Mostafa E. Rateb, Ahmed M. Sayed

**Affiliations:** 1 Department of Medical Laboratory Technology, Faculty of Applied Medical Sciences, King Abdulaziz University, Jeddah 21589, Saudi Arabia; hanialhadrami@kau.edu.sa; 2 Special Infectious Agent Unit, King Fahd Medical Research Centre, King Abdulaziz University, Jeddah 21589, Saudi Arabia; 3 Microbial Chemistry Department, National Research Centre, 33 El-Buhouth Street, Dokki, Giza 12622, Egypt; ahmedshalbio@gmail.com; 4 Department of Pharmacognosy, Faculty of Pharmacy, Beni-Suef University, Beni-Suef 62514, Egypt; abuh20050@yahoo.com; 5 Laboratory of Soil Biology, University of Neuchatel, 2000 Neuchatel, Switzerland; lassaad.belbahri@unine.ch; 6 School of Computing, Engineering & Physical Sciences, University of the West of Scotland, Paisley PA1 2BE, UK; 7 Department of Pharmacognosy, Faculty of Pharmacy, Nahda University, Beni-Suef 62513, Egypt

**Keywords:** MRSA, flavonoids, inverse virtual screening, PBP2a, bacterial resistance, adjuvant therapy

## Abstract

Recently, the interest in plant-derived antimicrobial agents has increased. However, there are no sufficient studies dealing with their modes of action. Herein, we investigate an in-house library of common plant-based phenolic compounds for their potential antibacterial effects against the methicillin-resistant *Staphylococcus aureus* (MRSA), a widespread life-threatening superbug. Flavonoids, which are considered major constituents in the plant kingdom, were found to be a promising class of compounds against MRSA, particularly the non-glycosylated ones. On the other hand, the glycosylated derivatives, along with the flavonolignan silibinin A, were able to restore the inhibitory activity of ampicillin against MRSA. To explore the mode of action of this class, they were subjected to an extensive inverse virtual screening (IVS), which suggested penicillin-binding protein 2a (PBP2a) as a possible target that mediates both the antibacterial and the antibiotic-synergistic effects of this class of compounds. Further molecular docking and molecular dynamic simulation experiments were conducted to support the primary IVS and the in vitro results and to study their binding modes with PBP2a. Our findings shed a light on plant-derived natural products, notably flavonoids, as a promising and readily available source for future adjuvant antimicrobial therapy against resistant strains.

## 1. Introduction

In 2018, the World Health Organization (WHO) announced that the number of antibiotic-resistant pathogenic bacteria had alarmingly increased to reach a perilous level that required a global cooperation [1]. About 2.8 million people are infected with antibiotic-resistant bacteria annually in the United States, with more than 35,000 dying as a result [2]. These superbugs are spreading globally, and the continuous emergence of new resistance mechanisms make our ability to treat common infectious diseases and some nosocomial infections a real challenge. The major cause behind this crisis is the misuse of our limited antibiotic resources in humans and animals. Moreover, the rate of new antibiotic development does not match the rate of growing resistance. Hence, besides improving the general awareness of proper antibiotics use, finding novel antibiotics should be a priority for a lot of research groups working in the field of drug discovery.

Most of the currently available antibiotics were derived from microbial sources, while the plant-based ones have been generally neglected, probably due to their weaker effects in comparison with the microbial-derived counterparts. With the increase in the exploration of plant-based antimicrobials in recent years, several potential candidates have shown promising activities that can be further developed into clinical therapeutics. However, the mode of action of most of these compounds is still unknown [3]. Penicillin-binding protein 2a (PBP2a) is one of the main proteins in Staphylococcus aureus that has evolved to resist β-lactam antibiotics [4]. Surprisingly, the active site of this protein can be modulated through another lateral binding site (i.e., allosteric site), and hence, the previously inactive β-lactam antibiotics can reach and inactivate this essential protein and, in turn, inhibit the superbug methicillin-resistant *Staphylococcus aureus* (MRSA) growth.

Recently, our research group has initiated an in-silico-based screening campaign to find out new possible drug leads that can be developed into new antimicrobial agents from different natural sources that are still considered the major source of successful antibiotics [5]. Consequently, we decided to continue our in-silico screening using an in-house plant-derived phenolic compounds library. We focused on the widely distributed and readily available plant phenolic metabolites during the preparation of this library so that we could find potential economic drug candidates that can be easily prepared from plants or even plant-waste products. PBP2a was proposed to be the most probable MRSA target for this library of compounds, depending on a comprehensive inverse virtual screening of the proteins hosted in the protein databank (PDB). In vitro testing (i.e., MIC and synergy study) further supported PBP2a as the possible target of this group of phenolic compounds, where targeting the active site led to direct growth inhibition, whereas targeting an allosteric site located 60 Å away from the active site led to a synergy effect with the β-lactam antibiotics.

**Figure 1:**
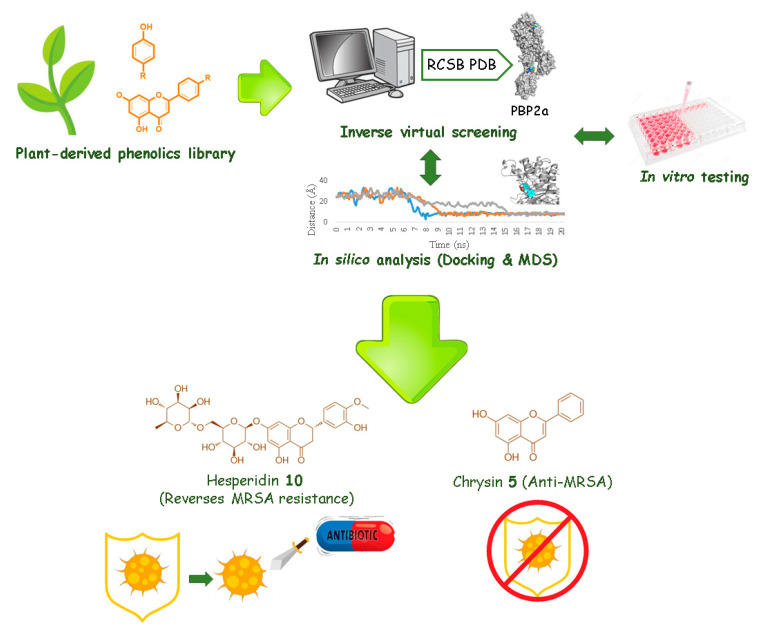
The applied strategy in the present investigation.

We believe that exploring natural products, notably those from plant origin, with the help of up-to-date computer-aided screening tools would eventually lead to a number of potential antimicrobial candidates that can help in the global urgency that, if not taken seriously, will lead us into a post-antibiotic era, in which common infections and minor injuries can easily lead to high mortality rates. The strategy applied in our current study is presented in Figure 1.

## 2. Results

### 2.1. In-Silico Screening

Finding an appropriate target for a given small bioactive molecule is a challenging issue in the drug discovery process. However, the recent advances in cheminformatics and computer-aided drug discovery and screening have significantly facilitated and shortened this phase of drug development. In this regard, we decided to utilize some of these approaches to suggest suitable MRSA targets for our library of phenolic-based compounds to further select some potential anti-MRSA candidates.

#### 2.1.1. Target Identification Using Inverse Virtual Screening

**Figure 2:**
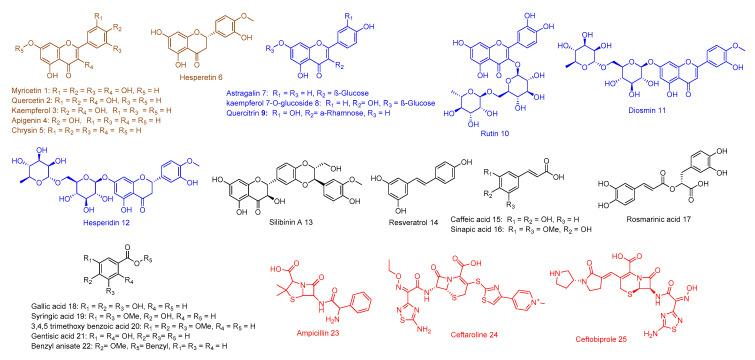
Compounds 1–22 of our phenolics-based library that were used in this study. This library consists of non-glycosylated flavonoids (brown color), glycosylated flavonoids (blue color), miscellaneous phenolic compounds (black color), in addition to ampicillin and the previously reported anti-MRSA agents (red color).

In order to characterize the possible antibacterial molecular target (s) for our phenolic compounds (1–22, Figure 2), we performed a large-scale structure-based inverse virtual screening against all protein targets hosted in the protein database (PDB) by using a publicly available docking server namely idTarget [6]. This strategy represents a fast and cheap step in detecting the most possible protein target for a given small molecule. Among the returned possible protein target, we selected the top-scoring MRSA-related ones.

Penicillin-binding protein 2a (PBP2a) appeared to be the most possible and common target for most of the flavonoids in our library (Tables S1 and S2), and hence it was selected for further in-silico investigation. Both DNA gyrase-B (Gyr-B), and D-alanine-D-alanine ligase (Ddl) were also predicted for a number of compounds in our library (3–4 compounds, Table S1) including four flavonoids (compounds 1–3,17). Interestingly, both Gyr-B and Ddl were previously reported to be inhibited by a number of flavonoids [7,8]. The virtual screening applied by this software depends on a rapid and rigid docking protocol. Hence, we dock the library again against the most possible protein target suggested in this step (i.e., PBP2a). 

#### 2.1.2. Docking Studies and Molecular Dynamic Simulation Refinement

In order to further support the primary inverse virtual screening step and directly analyze the molecular interactions with the predicted protein targets, the compounds library was subjected to a second round of flexible docking against the PBP2a active site using Autodock Vina, which is one of the best software used in finding the binding conformation efficiently [9]. Firstly, the accuracy of the docking protocol by Autodock Vina was tested by docking the co-crystalized ligands in the active sites of their corresponding proteins. The resulted docking poses showed confirmations similar to the co-crystallized ones with low root mean square deviation (RMSD = 0.6–1.7 Å), and the binding energy values ranged from −7.5 to −8.9 kcal/mol (Table S1). Consequently, we suggested the binding energy of −7.0 kcal/mol as a cut-off value in the subsequent docking experiments. 

Molecular docking scores sometimes do not reflect the ligand affinity [10,11], and thus top-scoring hits on some occasions failed to achieve good binding affinity and inhibition in the subsequent in vitro experiments and/or after molecular dynamic simulations (MDS). Hence, the ligand-protein complexes proposed by the docking experiments were thereafter validated by 20 ns MDS experiments. Furthermore, the main amino acid residues inside the protein’s binding site involved in the ligand-binding can be determined depending on the resulting MDS confirmations. Such important information may be utilized in further precise drug design (Table 1).

**Figure 3:**
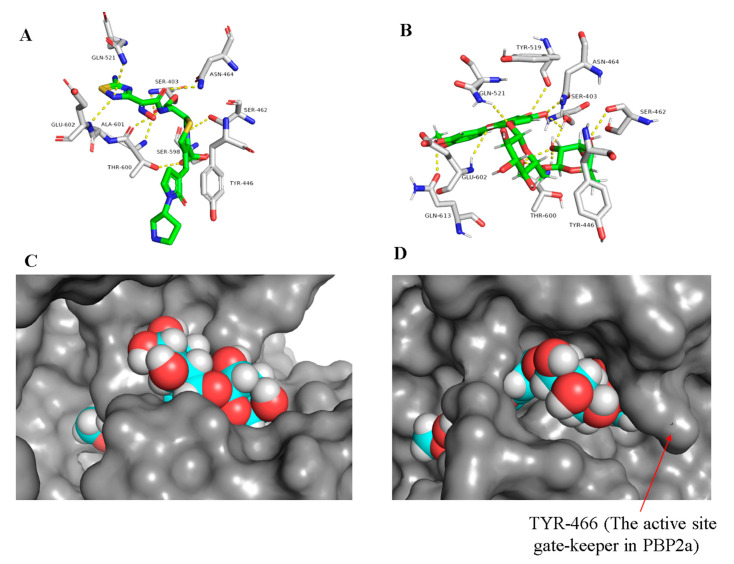
Binding mode of the co-crystalized ligand ceftobiprole (25) and diosmin (11) inside the PBP2a active site ((**A**,**B**), respectively), and docking of diosmin (11) inside PBPa and PBP2a ((**C**,**D**), respectively) to show the difference between the two proteins in terms of accessibility.

PBP2a is a modified form of D-alanyl-D-alanine transpeptidase that has evolved in the resistant Gram-positive bacterial strains (e.g., MRSA). The active site of this modified protein is highly conserved and closed by a tyrosine residue (TYR-466) which acts as a gate preventing the ligand (e.g., β-lactam antibiotics) from entering [12]. However, this closed state (Figure 3) is opened up under the control of an allosteric site located 60 Å away from the active site, and thus ligand-binding with this allosteric site can trigger a series of conformational changes in the whole protein that eventually leads to an accessible active site easily inhibited by β-lactam antibiotics [12]. Ceftaroline (24) was the first reported example of an MRSA-active β-lactam antibiotic that mediates its action through binding to the allosteric site to induce opening of the active site, and in turn, it can be covalently inhibited like other β-lactam antibiotics [12]. This protein was suggested to be a potential target for a number of flavonoid derivatives in our library (Table S1), particularly, the glycosylated ones (compounds 7–12, ΔG from −9.0 to −9.5 kcal/mol).

The docking results showed that the presence of a carbohydrate moiety in the flavonoid scaffold enabled it to establish an extensive network of strong H-bonds (11 H-bonds, <2.5 Å) with the active site’s amino acid residues. Additionally, they enabled the molecule to accommodate itself tightly inside the active site (e.g., diosmin (11), Figure 3B), and hence, all of these glycosylated derivatives got binding scores higher than that of the co-crystalized ligand (Table 1). However, these interesting docking results did not match with the in vitro anti-MRSA activity (MIC = 250 µg/mL, Table 2). Such a contradiction could be attributed to the presence of TYR-446 at the gate of the PBP2a active site (Figure 3), which hinders its accessibility by such large and highly polar molecules. Only small and relatively nonpolar compounds can access such hydrophobic narrow active site grooves (e.g., ceftobiprole (25), Figure 3A) [4,13]. MDS of a system-contained diosmin (11) near to the PBP2a active site (25 Å away from SER-403) supported this suggestion, where the polar carbohydrate moiety clashed with the hydrophobic gate-keeper residues (e.g., TYR-446) hindering its accessibility inside the active site groove. Additionally, these glycosylated flavonoids, in contrast to MRSA, showed moderate activity towards MSSA (MIC = 31.25 µg/mL, Table 2), where its PBPa active site is more exposed and shallower than its counterpart PBP2a and easily accessible by such a type of highly polar compounds (Figure 3C,D).

**Figure 4:**
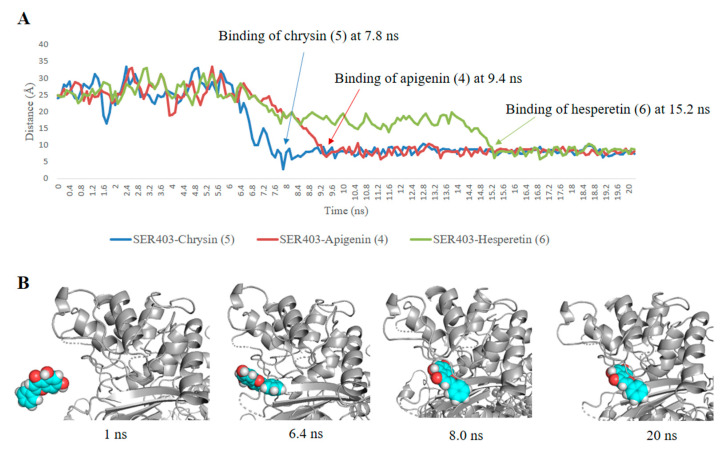
Binding process of compounds (4–6). (**A**) Distance changes between the *C_α_* atom of residue SER-403 (i.e., the catalytic residue inside PBP2a active site) and compounds 4–6. (**B**) Snapshots of PBP2a system during the binding process with chrysin (5).

**Figure 5:**
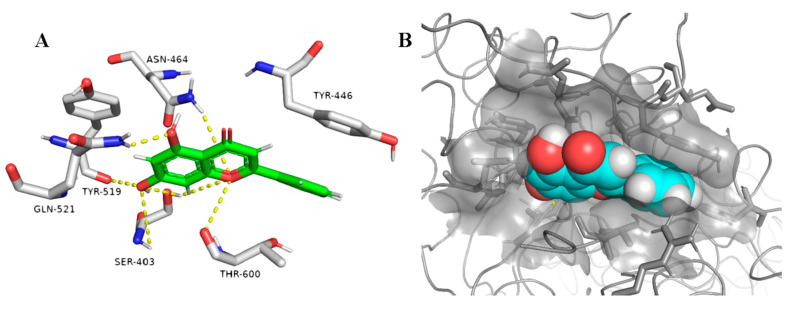
Binding mode of chrysin (5) inside PBP2a’s active site (**A**,**B**).

On the other hand, the non-glycosylated flavonoids (compounds 1–6) showed interesting fitting inside the PBP2a active site with binding free energy values ranging from −7.0 to −8.0 kcal/mol, while the highly hydroxylated derivative myricetin (1) showed relatively weaker binding affinity (*ΔG* = −5.8 kcal/mol). Regardless, the convergent binding affinity and interaction modes of the derivatives 1–6 showed different MIC values against MRSA ranged from 15.62 µg/mL for chrysin (5) to 125 µg/mL for myricetin (1) (Table 2). Being the smallest and the least hydroxylated (i.e., the least hydrophilic) molecule among its counterparts, particularly at ring B, chrysin (5) was able to easily access the PBP2a active site narrow groove during the 20 ns MDS (Figure 4A,B). Furthermore, compounds 4 and 6 were able to reach and bind to the active site, but they needed a longer time to do so, indicating that the hydroxylation at ring B decreased the accessibility toward the active site (Figure 4). Additionally, chrysin (5) was further subjected to another 20 ns MDS in its complexed form with the active site to study its stability and binding mode inside the active site (Figure 5, Figure S1). Interestingly, chrysin (5) was able to remain intact inside the narrow groove of the active site during the whole course of MDS, with a very small deviation from its starting position (RMSD = 0.9 Å). The binding mode study revealed that the non-hydroxylated ring B was a key player in its stabilization inside the active site, where it was sandwiched between THR-600 and TYR-446. In addition, the remaining part of the molecule (i.e., rings A and C) was involved in a network of H-bonds with the reported key amino acid residues (Figure 5) including the catalytic one (SER-403) [4]. Such structural information and the binding mode study can help in the design of more potent non-covalent inhibitors of PBP2a. 

**Figure 6:**
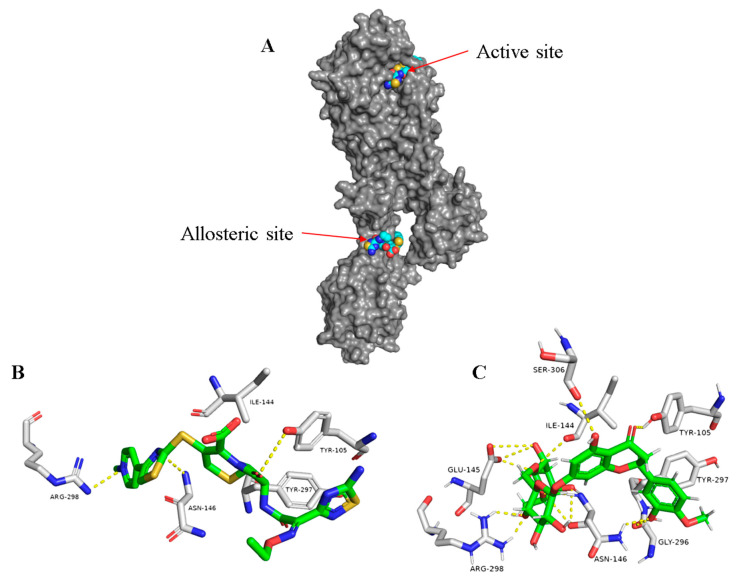
The whole structure of PBP2a showing both the active and allosteric sites bounded to ceftaroline (24) (**A**) and the binding modes of ceftaroline (24) and hesperidin (12) ((**B**,**C**), respectively) inside the allosteric site of PDB2a.

**Figure 7:**
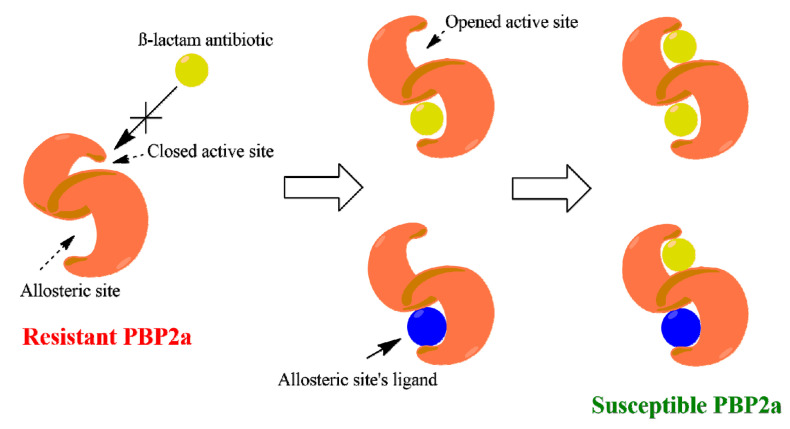
An illustration of the allosteric-mediated reversing of PBP2a resistance toward β-lactam antibiotics.

In another site on this interesting protein (i.e., PBP2a) and 60 Å away from the catalytic active site, the controller allosteric site is located (Figure 6A) [12]. This binding cavity in contrast to the active site is more exposed, and hence, glycosylated flavonoids can achieve good interaction with such an opened binding site. Docking results revealed that flavonoids attached to two sugar moieties (compounds 10–12), in addition to the flavonolignan silibinin A (13), showed interesting binding energy scores ranging from −8.8 to −10.3 kcal/mol. Moreover, these compounds displayed high stability inside the allosteric binding site during the MDS (e.g., hesperidin (12), Figure 6C and Figure S2), where they interacted with several amino acid residues, including the reported ones [12], through multiple strong H-bonds thanks to the polyhydroxyl groups in these compounds. In regard to the non-glycosylated flavonoids and the other phenolic derivatives, they could not get binding scores higher than −7.2 kcal/mol, and most of them did not achieve stable binding interactions inside the allosteric site during MDS (average RMSD > 6 Å). These findings reflect well on the anti-MRSA synergy study, where the glycosylated flavonoids significantly reduced the MIC values of ampicillin from 8- to 16-fold (Table 2). Such compounds most probably act in the same way as ceftaroline (24), where they bind to the allosteric receptor, which in turn induces a series of conformational changes ended by opening the active site to receive one molecule of ampicillin (Figure 7) [12].

### 2.2. In Vitro Inhibitory Activity

As a validation step to support the in-silico results, our in-house phenolics-based small library was screened for their inhibitory activity against both sensitive and resistant staphylococcal strains (Methicillin Sensitive *Staphylococcus aureus*-MSSA, and Methicillin-Resistant *Staphylococcus aureus*-MRSA). The results presented in Table 2 indicated that flavonoids (compounds 1–13) were the most active compounds in our plant phenolics-based library with MIC values ranged from 15.25 to 250 µg/mL. Both the glycosylated and the non-glycosylated flavonoids derivatives showed convergent inhibitory activity against MSSA (MIC = 31.25 µg/mL). On the other hand, the non-glycosylated derivatives were the most active group of compounds against MRSA with a decrease in the inhibitory activity upon the hydroxylation of this flavonoids scaffold, where the glycosylated derivatives were much less active or almost inactive on their own (MIC = 250 µg/mL).

In silico and in vitro results encouraged us to run an antibiotic synergistic study of these flavonoids with β-lactam antibiotic ampicillin (Table 2). The result of this study indicated that hesperetin (6), apigenin (4), and chrysin (5) have an additive effect with the β-lactam antibiotic ampicillin (FICI = 1). However, the glycosylated derivatives, rutin (10), diosmin (11), hesperidin (12), as well as the flavonolignan silibinin A (13) demonstrated interesting synergy with ampicillin despite their weak anti-MRSA activity (FICI = 0.31–0.625) and significantly improved the ampicillin effect on MRSA 8–16-fold. Both the inhibitory and synergistic activities against MRSA were in great accordance with the in-silico investigation that suggested PBP2a modulation as the possible mode of action of this class of plant-based compounds. 

### 2.3. Structure–Activity Relationship

It is obvious from our findings that the flavonoid (flavone and flavanone) scaffold is crucial for MRSA and its PBP2a inhibition. Simple flavones like chrysin (5) achieved the best inhibitory activity against MRSA (MIC = 15.62 µg/mL, Table 2) and the best stability inside the PBP2a’s active site (RMSD = 0.9 Å). The addition of hydroxyl groups in ring B (e.g., apigenin (4) and hesperetin (6) was associated with decreased anti-MRSA activity (MIC = 31.25 µg/mL). Additionally, it decreased the binding stability during the MDS (RMSD = 3.7 Å) and the binding free energy (Table 1). Moreover, the double bond between C-2 and C-3 appeared to be not an important feature for the bioactivity.

In case of the flavonol derivatives like myricetin (1), quercetin (2), and kaempferol (3), the hydroxylation at C-3 was associated with a further decrease in the anti-MRSA activity (MIC = 62.5–125 µg/mL) In addition, they were not able to access the active site during a 20 ns MDS. Hence, we can conclude that increasing the polarity of the flavonoid derivatives can decrease their accessibility to the active site and in turn its bioactivity. All the flavonol derivatives (compounds 1–3) showed little to no effect on the inhibitory activity of the β-lactam antibiotic ampicillin (i.e., indifferent effect, Table 2), while the flavone and flavanone derivatives (compounds 4–6) had an additive effect to ampicillin (Table 2).

**Figure 8:**
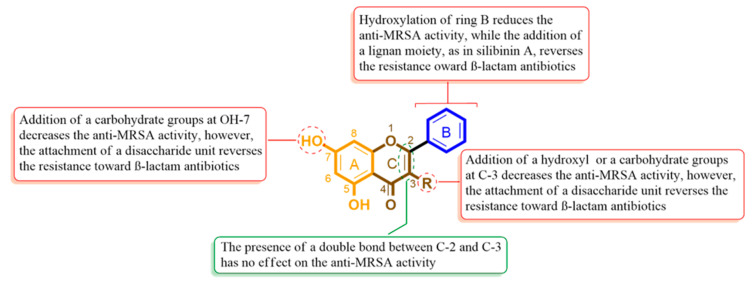
Structure-Activity Relationship (SAR) of the flavonoid scaffold indicates the possible structural features required for the inhibitory and synergistic effects of MRSA.

On the other hand, all the glycosylated flavonoid derivatives (compounds 10–12) together with the flavolignan silibinin A (13) showed very weak anti-MRSA activity which may be linked to their larger sizes and higher polarities that were driven from the disaccharide moiety at C-3 or the extinction at ring B, that in turn hindered their access to the closed PBP2a catalytic site. These unfavorable features for the active site enabled them to reverse the MRSA resistance toward ampicillin (i.e., showed a synergistic effect with FICI of 0.31 to 0.625, Table 2). Such an observation can be attributed to their high binding affinity and stability (Table 1) inside the PBP2a allosteric site in comparison with the other compounds. Figure 8 summarizes the structure-activity relationship (SAR) of these compounds. 

## 3. Discussion

Plants are a prolific producer of small molecules (>200,000 compounds) many of which exhibit antimicrobial activity (e.g., phenolics) [14]. However, their activity is generally weaker than that of common antibiotics produced by fungi and actinomycetes, thus they do not encourage further drug development [15]. Some plant metabolites such as flavonoids are produced at high levels to provide protection against harmful ultraviolet light. At the same time, they can serve as intrinsic defensive molecules that can protect plant tissues against pathogens [15]. Hence, such a class of compounds represents promising antibiotic candidates that can support our fight against the wide-spreading superbugs.

Despite there being dozens of plant-derived phenolics and flavonoids that show activity from a micromolar to sub-micromolar range, little is known about their mode of action [16]. Consequently, we aimed in the present investigation to shed light on the anti-MRSA potential of some selected wide-spread plant-derived phenolic metabolites, indicating their possible mode of action by applying an extensive in-silico analysis.

Most of the chosen phenolic compounds in our library are readily available from many edible plants. For example, hesperidin (12) and its aglycone hesperetin (6) present at high quantities in citrus fruits peels (e.g., orange and grapefruit peels) [17]. Rutin (10) and its aglycone quercetin (2) are also the main metabolites in many plants and their by-products such as blackberry, fenugreek, green tea, apricot, and olives [17]. Hence, the utilization of such natural products as therapeutic agents can provide an added value to most of the phenolic-rich edible plant, notably their processing waste products.

MRSA is one of the most dangerous spreading superbugs that is associated with a lot of nosocomial infections, which has modified most of their molecular targets of our currently available antibiotics. The modified transpeptidase PBP2a is considered the first-line defense tool of MRSA against its usual and specific β-lactams antibiotics, where its active site became closed and hardly accessible by this class of antibiotics. Surprisingly, this immune active site has a weak point of being under the control of another allosteric site in the same protein, and thus, targeting such a lateral binding site can make MRSA susceptible to their routine antibiotics once again (Figure 7). Previous reports have suggested a number of flavonoids as possible modifiers of MRSA cell wall and cell membrane [18,19,20]. Moreover, several polyhydroxylated flavonoids have demonstrated considerable synergistic effect with β-lactams antibiotics [16,21], and PBP2a has been suggested as the possible target [22].

Our inverse virtual screening applied in the present study proposes PBP2a to be the most possible target for the flavonoid class of compounds in our library. This in-silico suggestion is supported by a number of previous reports that illustrated flavonoids as possible modifiers of MRSA and other bacterial cell walls [18,19,20]. Further docking and molecular dynamic investigation not only confirmed the primary inverse virtual screening predictions but also revealed that some inactive derivatives can possibly modify the PBP2a allosteric site and hence, serve as adjuvants to reverse MRSA resistance toward β-lactams antibiotics (e.g., ampicillin 23). These findings were also in great accordance with the previous studies that highlighted polyhydroxylated flavonoids as promising synergistic agents to β-lactams antibiotics [16,21,23].

## 4. Materials and Methods 

### 4.1. Library Construction

All compounds used in this study (Figure 2) were purchased (compounds: 2,4,7, 6,11–15,17; Alfa Aesar, Massachusetts, USA and Sigma-Aldrich, Saint Louis, USA) or isolated from their natural source according to the previously reported procedures [24,25,26,27,28]. The constructed small library for the present study consisted of six non-glycosylated flavonoids (i.e., aglycones; 1–6), six glycosylated flavonoids (7–12), and other miscellaneous phenolic derivatives (13–22).

### 4.2. Bacterial Strains

We used in this study two standard strains of *Staphylococcus aureus*, one of them was susceptible to β-lactam antibiotics (i.e., methicillin sensitive *S. aureus*–SSA; ATCC 25923) and the other was resistant (methicillin resistant *S. aureus*–MRSA; ATCC 33591). Before the assays, both strains were cultured at 34 °C for 24 h. After culturing all strains on Mueller–Hinton agar, the cells were resuspended in Mueller–Hinton broth (MHB) (Oxoid^®^) to give 10^8^ colony-forming units/mL [29].

### 4.3. Minimum Inhibitory Concentration (MIC) Assay 

MIC values were determined for each compound in the library according to the broth microdilution assay [5]. Briefly, test solutions were made by dissolving each tested compound in DMSO (Sigma, Milan, Italy). Several colonies of each tested bacterial strain were inoculated in 10 mL of sterilized MHB and then incubated for 24 h at 37 °C. Afterward, each bacterial suspension was adjusted to a final concentration of 100 cfu/mL (OD600 nm); then 100 µL of the bacterial suspension were added in the 96-well cell culture plate (Cellstar^®^, Greiner Bio-One, Frickenhausen, Germany), with a serially diluted (1:2) test compound solutions. The optical density (600 nm) of every well was measured using a Spectrostar Nano Microplate Reader (LABTECH GmbH, Allmendgrun, Germany). The lowermost compound concentration that inhibits the bacterial growth after 24 h of incubation corresponded to the MIC. Ampicillin (Sigma Aldrich, Saint Louis, USA) was used as a reference standard.

### 4.4. Checkerboard Microdilution Assay

To study the possible anti-MRSA synergy of the tested compounds with the β-lactam antibiotic, we determined the lowest concentration of both the tested compound and ampicillin in combination that required to inhibit MRSA growth by the checkerboard microdilution assay [30]. The starting concentration of the used bacterial suspension was 1.5 × 10^5^ CFU mL^−1^. The results were recorded after the incubation of cultured plates at 37 °C for 24 h. To determine the synergistic, additive, indifferent or antagonistic effects of the tested compounds and the antibiotic, fractional inhibitory concentration indices (FICIs) were determined [30,31]. The following formulas were applied to calculate the fractional inhibitory concentration (FIC) index: FIC of drug A = MIC of drug A in combination/MIC of drug A alone; FIC of drug B = MIC of drug B in combination/MIC of drug B alone; and FIC index = FIC of drug A + FIC of drug B. The effect of the tested compound in combination with the antibiotic was considered synergistic when the FICI < 1, additive when the FICI = 1, indifferent when the FICI ≤ 2, and antagonistic when the FICI > 2 [31].

### 4.5. Inverse Virtual Screening

In order to identify the possible molecular targets that can mediate the observed anti-MRSA activity, we subjected the library’s compounds to an inverse docking approach [32] using a cloud-computing platform, namely idTarget [6] that can screen the query molecules against all proteins hosted in the protein data bank (https://www.rcsb.org/). Briefly, the 3D structure of each tested compound was prepared in pdb format and submitted to the idTarget server using the “fast mode” option, and the other computational parameters were set to a default value. idTarget automatically removes all ligands and/or cofactors from the proteins before performing the reverse docking procedure. The top MRSA targets retrieved by idTarget were then selected (Table S1). MRSA PBP2a (PDB codes: 4DKI, 6Q9N, 3ZFZ) was the top-scoring protein for most of the library’s compounds, and hence, it was selected for further docking, molecular dynamic simulation (MDS), and structure-activity relationship studies. 

### 4.6. Molecular Docking

To further validate the inverse docking results and investigate the binding interactions inside the active and allosteric sites of PBP2a, the tested compounds were subjected to molecular docking using AutoDock Vina [33]. MRSA PBP2a crystal structure with PDB codes of 4DKI and 3ZFZ were used for docking experiments. In regard to MSSA PBPa, we used the protein structure with PDB code of 3UPO. The applied docking protocol deals with the protein as a rigid structure and the tested compound as a flexible structure during its computations. The co-crystallized ligands were utilized to determine the binding sites. The ligand-to-binding-site shape matching root mean square (RMSD) threshold was set to 2.0 Å. The interaction energies were determined using the Charmm Force Field (v.1.02) with 10.0 Å as a non-bonded cutoff distance and distance-dependent dielectric. Then, 5.0 Å was set as an energy grid extending from the binding site. The tested compounds were energy-minimized inside the selected binding pocket. The editing and visualization of the generated binding poses were performed using Pymol software [34].

### 4.7. Molecular Dynamic Simulation

Molecular dynamic simulations (MDS) for ligand–enzyme complexes were performed using the Nanoscale Molecular Dynamics (NAMD) 2.6 software [35], applying the CHARMM27 force field [36]. Hydrogen atoms were added to the protein structures using the psfgen plugin included in the Visual Molecular Dynamic (VMD) 1.9 software [37]. Afterward, the whole systems were solvated using TIP3P water particles and 0.15 M NaCl. The energy of the generated systems was firstly minimized and gradually heated to 300 K and equilibrated for 200 pseconds. Subsequently, the MDS was continued for 20 ns, and the trajectory was stored every 0.1 ns and further analyzed with the VMD 1.9 software. The MDS output were sampled every 0.1 ns to evaluate the conformational changes of the entire system to analyze the root mean square deviation (RMSD) and root mean square fluctuation (RMSF). The topologies and parameters of the tested compounds were prepared using the VMD Force Field Toolkit (ffTK) [37] and the online software Ligand Reader & Modeler (http: //www.charmm-gui.org/?doc=input/ligandrm) [38]. Binding free energies were calculated using a neural-networking-based software, namely K*_DEEP_* (https://www.playmolecule.org/Kdeep/) [39].

## 5. Conclusions

In conclusion, on the basis of our in-silico analysis that was supported by in vitro outcomes together with the SAR study presented in this study, we believe that this class of plant metabolites is considered promising as anti-MRSA therapeutics and can serve as a potential starting point for further development of more potent plant-based antibiotics or adjuvant therapy.

## Figures and Tables

**Table 1:** Binding energies (in kcal/mol) of compounds 1–13 retrieved for both the active and allosteric sites together with their main molecular interactions.

Ligand	∆G*_Vina_* (Active and Allosteric Sites)	∆G * *_KDEEP_* (Active and Allosteric Sites)	Hydrogen Bonding Interactions		Hydrophobic Interactions	
			Active Site	Allosteric Site	Active Site	Allosteric Site
Myricetin (1)	>−7.0, −7.0	>−7.0, −6.8	ASN-464, TYR-519, GLN-521	TYR-105, ASN-146	TYR-446	TYR-297
Quercetin (2)	−7.2, −7.2	<−7.0, −7.1	ASN-464, TYR-519, GLN-521	TYR-105,TYR-297	TYR-446	-
Kaempferol (3)	−7.6, <−7.0	<−7.0, <−7.0	SER-403 GLN-521	ASN-146, ILE-144	TYR-446	-
Apigenin (4)	−8.0, <−7.0	−7.8, <−7.0	SER-403, ASN-464, TYR-519, GLN-521	ARG-298, GLY-296	TYR-446	-
Chrysin (5)	−7.9, <−7.0	−8.0, <−7.0	SER-403, ASN-464, TYR-519, GLN-521, THR-600	GLU-145, ASP-295	TYR-446	TYR-297
Hesperetin (6)	−7.8, −7.2	−8.0, <−7.0	SER-403, ASN-464, TYR-519, THR-600	ILE-144, ASP-295	TYR-446	TYR-297
Astragalin (7)	−9.0, −8.1	−8.7, −8.5	SER-403, ASN-464, TYR-519, THR-600, GLN-613	ILE-144, GLU-145, ASN-146, GLY-296	-	TYR-105
Kaempferol 7-*O*-glucoside (8)	−9.1, −7.9	−8.7, −8.4	SER-403, SER-462, ASN-464, TYR-519	TYR-105, ILE-144, GLU-145, ASN-146	-	TYR-297
Quercitrin (9)	−9.0, −8.1	−8.8, −8.3	SER-403, ASN-464, TYR-519, THR-600, GLN-613	ILE-144, GLU-145, ASN-146, GLY-296	-	TYR-105
Rutin (10)	−9.4, −8.8	−9.0, −8,7	SER-403, ASN-464, TYR-519, THR-600, GLN-613	TYR-105, ILE-144, GLU-145, ASN-146, GLY-296	-	-
Diosmin (11)	−9.6, −9.8	−9.9, −9.2	SER-403, SER-462, ASN-464, TYR-519, THR-600, GLN-613	TYR-105, ILE-144, GLU-145, ASN-146, GLY-296	-	-
Hesperidin (12)	−9.5, −10.3	−9.6, −9.9	SER-403, SER-462, ASN-464, TYR-519, THR-600, GLN-613	TYR-105, ILE-144, GLU-145, ASN-146, GLY-296, ARG-298, SER-306	-	TYR-297
Silibinin A (13)	−8.8, −9.5	−8.9, −9.3	SER-403, SER-462, ASN-464, TYR-519, THR-600	TYR-105, ILE-144, GLU-145, ASN-146, ASP-295, GLY-296, ARG-298, SER-306	TYR-446	TYR-105, TYR-297
Ampicillin (23)	>−7.0, >−7.0	>−7.0, −7.1	SER-462, ASN-464, TYR-519	ILE-144, GLU-145, ASN-146	-	TYR-297
Co-crystalized ligands (24 and 25)	−8.9 ^a^, −9.1 ^b^	−8.5 ^a^, −9.0 ^b^	SER-403 ^#^, SER-462, ASN-464, GLN-521, SER-598, AlA-601 GLU-602, THR-600	TYR-105, ILE-144, ASN-146, ARG-298	TYR-446	TYR-297

* Binding free energy calculated by a neural networking method (K*_DEEP_*). ^a^ Ceftobiprole (25), ^b^ Ceftaroline (24), ^#^ covalent interaction.

**Table 2:** Inhibitory activity of the tested compounds alone and in combination with ampicillin against both MSSA and MRSA.

Tested Compound	MIC MSSA (µg/mL)	MIC MRSA (µg/mL)	FICI *	Inference
Myricetin (1)	31.25	125	2	Indifferent
Quercetin (2)	31.25	62.5	1.5	Indifferent
Kaempferol (3)	31.25	62.5	1.5	Indifferent
Apigenin (4)	15.62	31.25	1	Additive
Chrysin (5)	15.62	15.62	1	Additive
Hesperetin (6)	15.62	31.25	1	Additive
Astragalin (7)	31.25	250	1.5	Indifferent
kaempferol 7-*O*-glucoside (8)	31.25	250	1.5	Indifferent
Quercitrin (9)	31.25	250	1.5	Indifferent
Rutin (10)	125	250	0.625	Synergistic
Diosmin (11)	31.25	250	0.31	Synergistic
Hesperidin (12)	31.25	250	0.31	Synergistic
Silibinin A (13)	62.5	250	0.31	Synergistic
Resveratrol (14)	125	250	1.5	Indifferent
Caffeic acid (15)	>250	250	1.5	Indifferent
Sinapic acid (16)	125	>250	2	Indifferent
Rosmarinic acid (17)	>250	>250	2	Indifferent
Gallic acid (18)	>250	>250	2	Indifferent
Syringic acid (19)	>250	>250	2	Indifferent
Trimethoxy benzoic acid (20)	>250	>250	2	Indifferent
Gentisic acid (21)	>250	>250	2	Indifferent
Benzyle anisate (22)	>250	>250	2	Indifferent
Ampicillin (23)	0.25	125	-	-

* Fractional inhibitory concentration index (FICI) was calculated for the synergy between compounds (1-22) and ampicillin toward MRSA.

## Supplementary Materials

The following are available online at https://www.mdpi.com/2079-6382/9/9/562/s1, Figure S1: RMSDs of PBP2a-ligand complexes and the ligands. (**A**) apigenin (4), (**B**) chrysin (5), (**C**) hesperetin (6); Figure S2: RMSDs of PBP2a-ligand complexes and the ligands. (**A**) rutin (10), (**B**) diosmin (11), (**C**) hesperidin (12), (**D**) silibinin A (13). Table S1: List of the potential anti-MRSA targets suggested by idTarget, Table S2: List of compounds that predicted to be possible inhibitors for certain *Staphylococcal* protein target.
