# Supplementary material for: Flavonoids as Potential anti-MRSA Agents through Modulation of PBP2a: A Computational and Experimental Study
Source: Antibiotics (Basel). 2020 Aug 31;9(9):562. doi: 10.3390/antibiotics9090562 (PMC7559925; doi:10.3390/antibiotics9090562)
Supplement: Supplementary file 1 [file antibiotics-09-00562-s001.pdf]

Supplemental Material

Flavonoids as Potential anti-MRSA Agents through Modulation of PBP2a: A Computational and Experimental Study.

Hani A. Alhadrami [1,2], Ahmed A. Hamed [3], Hossam M. Hassan [4], Lassaad Belbahri [5], Mostafa E. Rateb [6, *], Ahmed M. Sayed [7, *]

[1] Faculty of Applied Medical Sciences, Department of Medical Laboratory Technology, King Abdulaziz University, P. O. Box 80402, Jeddah, 21589, Kingdom of Saudi Arabi,

[2] King Fahd Medical Research Centre, King Abdulaziz University, P. O. Box 80402, Jeddah, 21589, Kingdom of Saudi Arabia, hanialhadrami@kau.edu.sa (HAA)

[3] Microbial Chemistry Department, National Research Centre, 33 El-Buhouth Street, P.O. Box 12622, Dokki, Giza, Egypt, ahmedshalbio@gmail.com (AAH)

[4] Department of Pharmacognosy, Faculty of Pharmacy, Beni-Suef University, Beni-Suef 62514, Egypt, abuh20050@yahoo.com (HMH)

[5] Laboratory of Soil Biology, University of Neuchatel, 2000 Neuchatel, Switzerland, lassaad.belbahri@unine.ch (LB)

[6] School of Computing, Engineering & Physical Sciences, University of the West of Scotland, Paisley PA1 2BE, UK,

[7] Department of Pharmacognosy, Faculty of Pharmacy, Nahda University, Beni-Suef 62513, Egypt.

* Correspondence: Mostafa.Rateb@uws.ac.uk (MER) and Ahmed.Mohamed.Sayed@nub.edu.eg (AMS).

Table S1. List of the potential anti-MRSA targets suggested by idTarget.

Target name	Target PDB ID	Predicted for	Biological activity	ΔG_{Vina} kcal/mol*
Penicillin-Binding Protein 2a (PBP2a)[a]	4DKI, 6Q9N, 3ZFZ	Compounds: 2-11, 17	Cell wall synthesis	-8.9, -8.8, -7.5
D-alanine-D-alanine ligase (Ddl)	3N8D	Compounds :1, 2, 14	Cell wall synthesis	-9.6
DNA gyrase-B (Gyr-B)	5D6P	Compounds: 1, 2, 3, 7-9	Negative supercoiling of the DNA	-9.2

*Binding energy scores of the co-crystalized ligands of each protein after the re-docking by Audodock Vina.

[a]PBP2a was selected as the most possible target because it was the best-predicted protein (predicted for 10 compounds of 20), particularly, the flavonoids.

Table S2. List of compounds that predicted to be possible inhibitors for certain Staphylococcal protein target.

Compound name	ΔG* kcal/mol	ΔG_{Vina}** kcal/mol (Active site)	ΔG_{Vina}** kcal/mol (Allosteric site site)	Chemical class	Subclass
Myricetin (1)	-	< -7.0	-7.0	Phenolics	Flavonoid
Quercetin (2)	-7.1	-7.2	-7.2	Phenolics	Flavonoid
Kaempferol (3)	-7.8	-7.6	< -7.0	Phenolics	Flavonoid
Apigenin (4)	-7.8	-8.1	< -7.0	Phenolics	Flavonoid
Chrysin (5)	-7.0	-7.9	< -7.0	Phenolics	Flavonoid
Hesperetin (6)	-7.3	-7.8	-7.2	Phenolics	Flavonoid
Astragalin (7)	-8.3	-9.0	-8.2	Phenolics	Flavonoid
kaempferol 7-*O*-glucoside (8)	-8.2	-9.1	-7.9	Phenolics	Flavonoid
Quercitrin (9)	-8.3	-9.0	-8.1	Phenolics	Flavonoid
Rutin (10)	-8.2, (-7.9)#	-9.4	-8.8	Phenolics	Flavonoid
Diosmin (11)	-8.5, (-9.1) #	-9.6	-9.8	Phenolics	Flavonoid
Hesperidin (12)	-8.4, (-9.5) #	-9.5	-10.3	Phenolics	Flavonoid
Silibinin A (13)	-8.0	-8.8	-9.5	Phenolics	Flavonolignan
Resveratrol (14)	> -7.0	> -7.0	> -7.0	Phenolics	Stilbene
Caffeic acid (15)	> -7.0	> -7.0	> -7.0	Phenolics	Cinnamic acid derivative
Sinapic acid (16)	> -7.0	> -7.0	> -7.0	Phenolics	Cinnamic acid derivative
Rosmarinic acid (17)	-7.2	> -7.0	> -7.0	Phenolics	Cinnamic acid derivative
Gallic acid (18)	> -7.0	> -7.0	> -7.0	Phenolics	Benzoic acid derivative
Syringic acid (19)	> -7.0	> -7.0	> -7.0	Phenolics	Benzoic acid derivative
Trimethoxy benzoic acid (20)	> -7.0	> -7.0	> -7.0	Phenolics	Benzoic acid derivative
Gentisic acid (21)	> -7.0	> -7.0	> -7.0	Phenolics	Benzoic acid derivative
Benzyle anisate (22)	> -7.0	> -7.0	> -7.0	Phenolics	Benzoic acid derivative

*Binding energy predicted by idTarget.

**Binding energy predicted by Autodock Vina.

Binding energy predicted by idTarget for the allosteric site

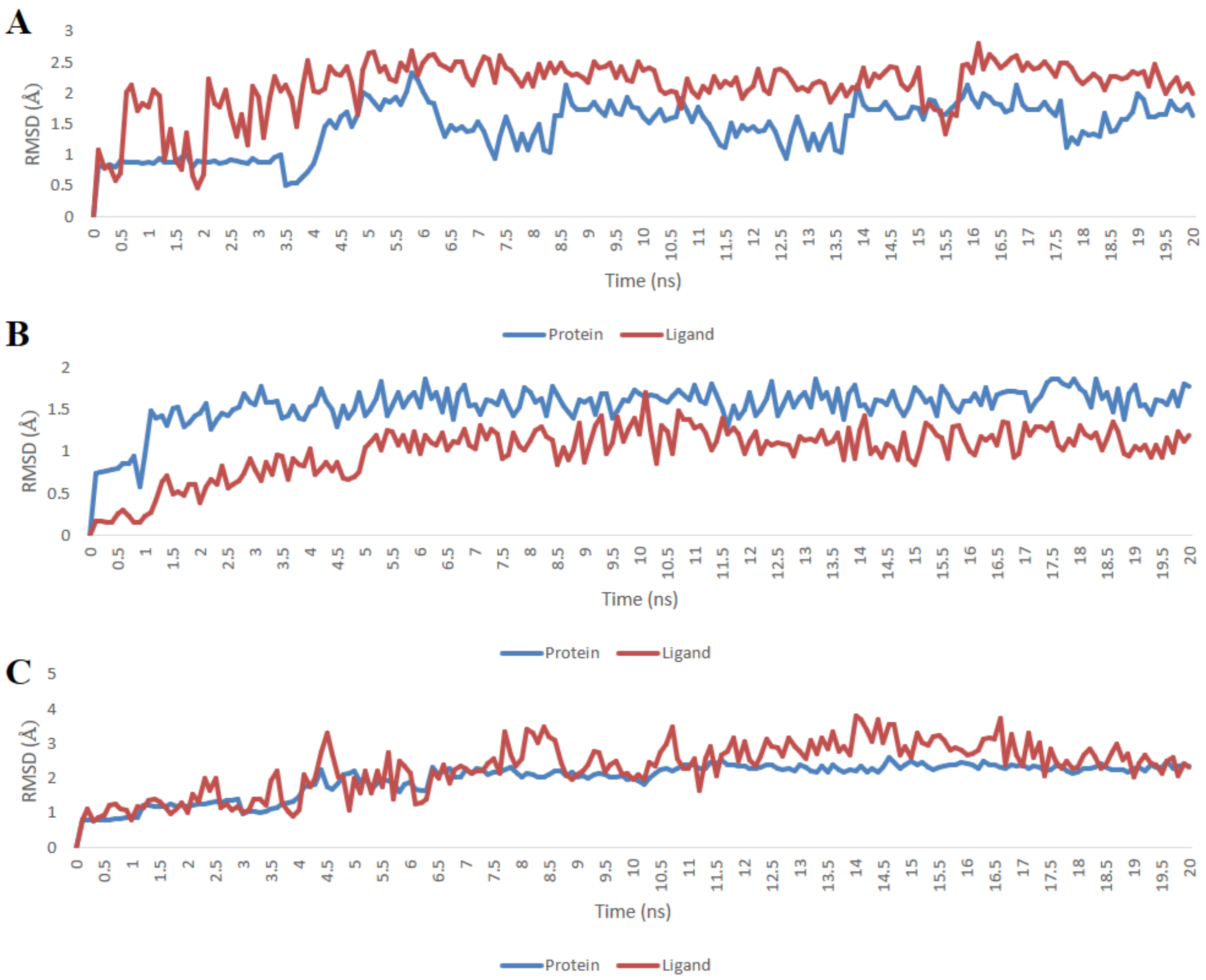

Figure S1. RMSDs of PBP2a-ligand complexes and the ligands. (**A**) Apigenin (4), (**B**) Chrysin (5), (**C**) Hesperetin (6).

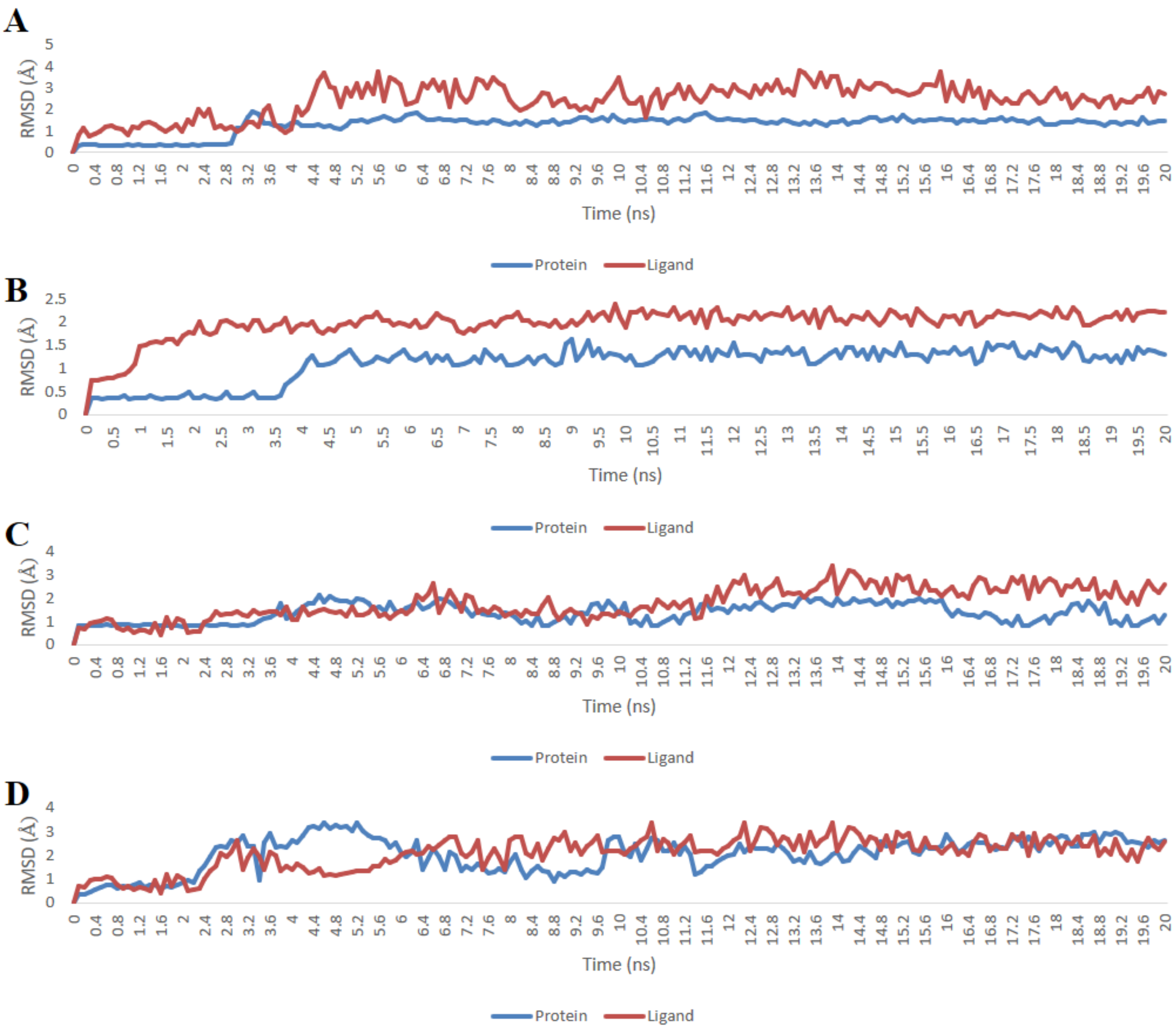

Figure S2. RMSDs of PBP2a-ligand complexes and the ligands. (**A**) Rutin (10), (**B**) Diosmin (11), (**C**) Hesperidin (12), (**D**) Slibinin A (13).
